# Vertex Assignment for Frequency Chaos Game Representation of Proteins Affects Classification Performance

**DOI:** 10.34133/csbj.0225

**Published:** 2026-09-14

**Authors:** Bálint Biró, Tímea Subicz, Róbert Barta, Soma Sándor Hartai, László Hiripi, Orsolya Ivett Hoffmann

**Affiliations:** ^1^ Group BM, Data Insights Team, _VOIS, Budapest, Hungary.; ^2^ Department of Animal Biotechnology, Institute of Genetics and Biotechnology, Hungarian University of Agriculture and Life Sciences, Gödöllő, Hungary.; ^3^ Laboratory Animal Science Coordination Center, Semmelweis University, Budapest, Hungary.

## Abstract

Chaos game representation and its frequency matrix variant are popular sequence encodings commonly assumed to be invariant to alternative nodal orientations. In this paper, we systematically test this assumption on 3 protein benchmark datasets with 2 convolutional neural networks and show that different vertex assignments lead to significant differences in classification performance. Evaluating 1,000 random encodings revealed a wide performance range, proving that vertex assignment is a critical parameter rather than an arbitrary choice. Furthermore, in most of the cases, frequency chaos game representation-based data augmentation proved to be effective only under moderate settings, while aggressive augmentation degraded performance due to distributional shifts introduced by permuted encodings.

## Introduction

**Fig. 1.**
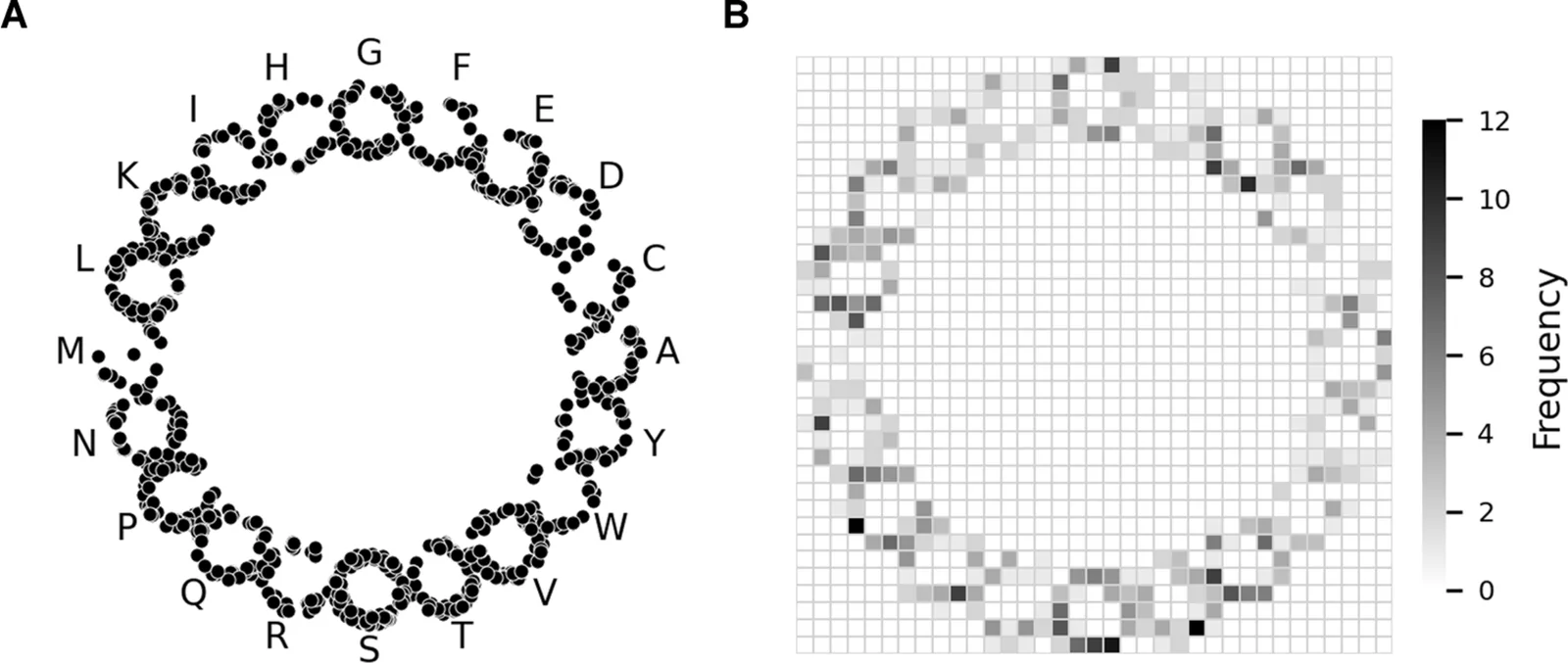
Sample images of CGR (A) and FCGR (B) representation of P20273 human B-cell receptor CD22 sequence.

In recent years, chaos game representation (CGR) and the frequency matrix of CGR (FCGR) (Fig. 1) emerged as encoding techniques that can capture both local and global compositional heterogeneities of sequential data. CGR is considered as a graphical method that employs an iterated function system (IFS) that transforms 1-dimensional sequential data into a fractal within 2-diemnsional (2D) space [1,2]. Initially, these IFSs were interpreted as a set of linear equations, one per dimension [3]. A simplified interpretation was adopted and is presented in the following section. These encoding techniques quickly gained popularity in the biological research community for various tasks including functional analysis of nucleic acid sequences [1,4,5], protein sequences [2,6], and phylogenetic experiments [7,8]. CGR/FCGR-based approaches became especially widespread coupled with convolutional neural networks (CNNs) [1,2,4–7,9,10] as CNNs became popular in image-related tasks.

To formalize these encoding procedures, we briefly introduce the mathematical background of CGR/FCGR applied to protein sequences [Eqs. (1) to (6)]. Let AA={A,C,D,E,F,G,H,I,K,L,M,N,P,Q,R,S,T,V,W,Y} denote the ordered set of proteinogenic amino acids and $S=S_{1},S_{2}\cdots S_{n}$ be a protein sequence with a property of $S_{i}\in \mathrm{AA}$. Then, the CGR encoding of this sequence is the 2-dimensional representation of the ordered pairs $\left(x_{i},\;y_{i}\right)$, which can be defined iteratively as

$$\begin{array}{c} (x_{i},\;y_{i})=\mathrm{sf}\;\left[(x_{i-1},\;y_{i-1})+p\left(s_{i}\right)\right],\text{if}\;i\geq 1; \end{array}$$ (1)

where

$$\left(x_{0},\;y_{0})=(0,0\right)$$ (2)

$$\begin{array}{c} p\left(s_{i}\right)\left\{\begin{array}{c} (\text{cos}\frac{2\pi *1}{\left|\mathrm{AA}\right|},\;\text{sin}\frac{2\pi *1}{\left|\mathrm{AA}\right|}),s_{i}={\mathrm{AA}}_{1}=A\\ \vdots \\ (\text{cos}\frac{2\pi *20}{\left|\mathrm{AA}\right|},\;\text{sin}\frac{2\pi *20}{\left|\mathrm{AA}\right|}),s_{i}={\mathrm{AA}}_{20}=Y \end{array}\right. \end{array}$$ (3)

$$\begin{array}{c} \mathrm{sf}=1-\frac{\text{sin}\left(\frac{\pi }{\left|\mathrm{AA}\right|}\right)}{\text{sin}\left(\frac{\pi }{\left|\mathrm{AA}\right|}\right)+\text{sin}\left(\frac{\pi }{\left|\mathrm{AA}\right|}+2\left(\frac{m}{\left|\mathrm{AA}\right|}\right)\right)} \end{array}$$ (4)

where $m=\left\lfloor \frac{\left|\mathrm{AA}\right|}{4}\right\rfloor$. The sf=0.865 setting is responsible for minimizing the overlap in the CGR, hence improving computational performance [2,11]. In this case, the FCGR is a $2^{k}\times 2^{k}$ matrix $F=\left(a_{i,j}\right),\leq i,j\leq 2^{k},i,j\in \mathbb{N}$. There is an element, $a_{i,j}$ for each k-mer $b\in {\mathrm{AA}}^{k}$ in the matrix F that corresponds to the number of occurrences of b as a subsequence of S. Because the FCGR forms an image-like representation of the sequence, it can be directly used as input for CNNs to learn spatial patterns. The position of each element, $a_{i,j}$ in the matrix is defined as:

$$i=2^{k}-\left[2^{k-1}\left(x+1\right)\right]+1$$ (5)

$$j=\left[2^{k-1}\left(y+1\right)\right]$$ (6)

where (x,y) is the CGR encoding of the k-mer b. The number of possible vertex assignments can be defined as $P=\frac{(\left|\mathrm{AA}\right|-1)\;!}{2}$.

Even though alternative nodal orientations would result in unique representations, a fraction of the scientific literature—mostly in phylogenetics studies—pays attention to vertex assignment for generating CGRs [8,12]. Some hypothesize that those permuted representations would yield similar performances [13]. Hence, using CGR/FCGR encodings with different vertex assignments would serve as a meaningful approach for data augmentation [14]. Nevertheless, as to our current knowledge, no systematic analysis was performed to assess the influence of different nodal orientations for CGR/FCGR on the performance of classification models. Therefore, 2 hypotheses were formulated: (a) CNNs trained on FCGRs derived from CGRs with different vertex assignments would achieve similar performance, and (b) CNNs trained on FCGRs derived from augmented CGRs (with different vertex assignments) would result in a performance similar to or better than that of CNNs trained on nonaugmented data.

## Materials and Methods

DeepLoc [15], ImmunoDB [16], and PFAM [17] benchmark datasets were used. Sequences containing non-proteinogenic amino acids were removed, resulting in 6,892, 3,964, and 9,548 sequences, respectively. From PFAM, the first 5 most abundant classes were used to reduce dataset size, hence computational requirements. The datasets were chosen to ensure heterogeneity in the underlying tasks (Table 1).

**Table 1.** Per-class details of the benchmark datasets

Dataset	Classes	Instances per classes
DeepLoc [15]	Soluble	4,086
	Membrane	2,806
ImmunoDB [16]	Immunogenic	2,078
	Nonimmunogenic	1,886
PFAM [17]	Hydrolase	1,980
	Hydrolase inhibitor	1,667
	Immune system	1,940
	Isomerase	1,984
	Lyase	1,977

To evaluate different vertex assignments for CGR/FCGR, 1,000 randomly encoded FCGRs were generated from the benchmarks using the kaos R package [10] with a resolution of 35. FCGRs were Max-Abs normalized.

Custom lightweight CNN and ResNet18 [18] models were implemented in PyTorch [19] using Skorch [20]. The Custom CNN consisted of a single 2D convolutional layer (3 × 3 kernel) followed by ReLU activation and 2 × 2 max pooling. The resulting feature maps were flattened and passed to a fully connected output layer. The networks were trained using the Adam optimizer (learning rate: 0.001) and the cross entropy loss. These models were trained on the previously mentioned FCGRs. Using a fixed train/test split with 75:25 ratio, the F1 score metric on the test set was recorded across all the experiments. For the multiclass benchmarks, macro averages of F1 scores were used.

To quantify training noise, 1,000 trainings with varying seeds with each of the benchmarks were performed using one randomly chosen encoding. Then, the F1 scores on the test sets were compared to the F1 scores of the 1,000 trainings on the randomly encoded FCGRs.

Min, Q1, Q2, Q3, and Max dedicated encodings were selected based on F1 score ranking. Stratified 10 fold cross-validation (CV) was implemented in Scikit-Learn [21].

To assess FCGR-based data augmentation, the Min encoding across benchmark datasets was augmented for training, either with a random sample from all 4 other encodings pooled together (mix strategy) or using one alternate encoding at a time (single strategy). Then, a hold out, fixed test set (25%) from the Min encoding was used to test a CNN. During these augmentation experiments, original dataset ratio (ODR) was varied to see whether slight or more extreme augmentation can be applied without a significant drop in the metric recorded.

The statistical analysis was performed with SciPy [22] and Statsmodels [23]. Kruskal–Wallis test was applied with Benjamini–Hochberg false discovery rate correction for multiple testing if needed. *P* values less than 0.05 were considered as significant.

Source codes can be found at balintbiro/CGR_representation.

## Results

**Fig. 2.**
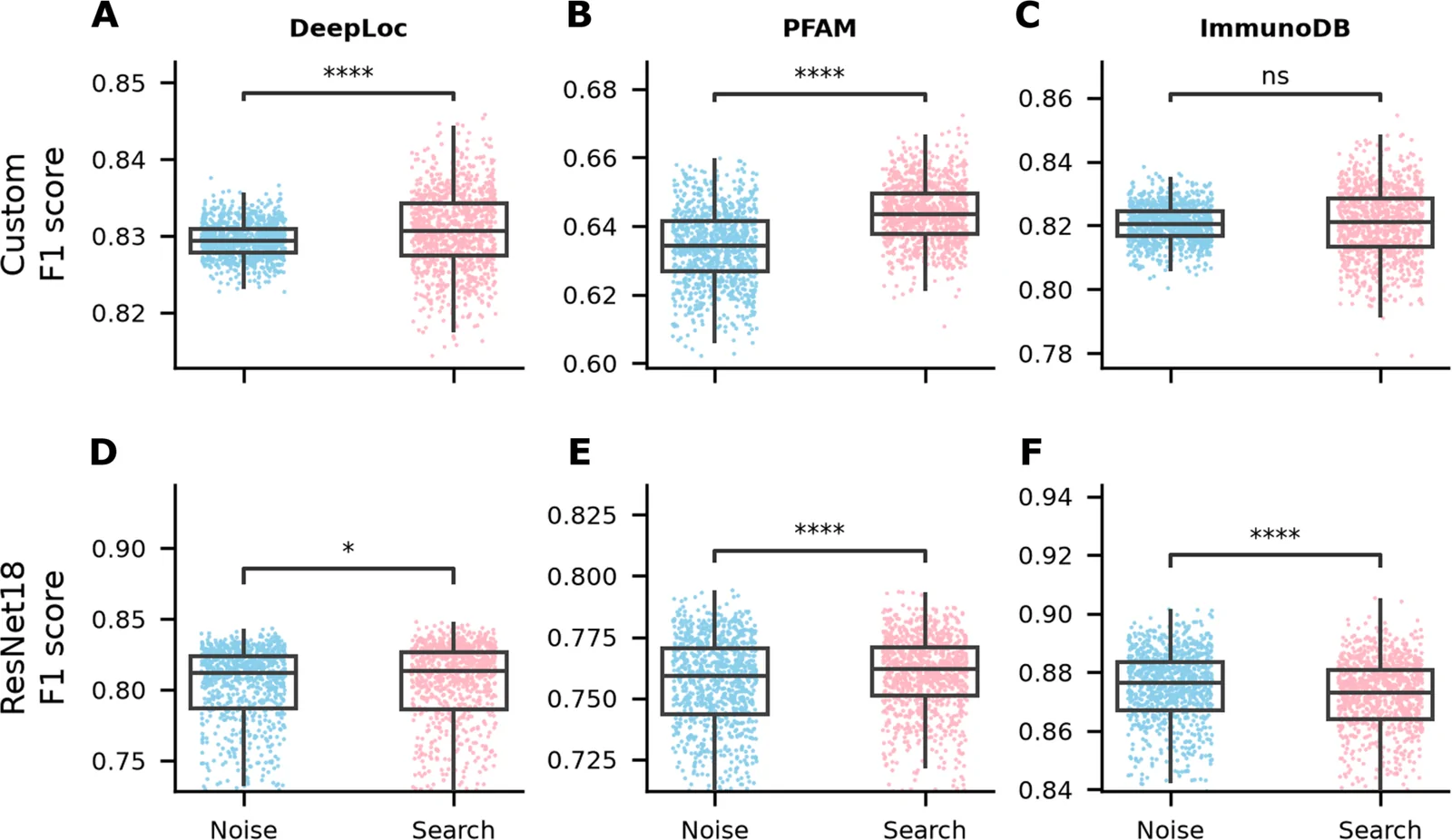
Training noise quantification. (A to C) Training noise across datasets using Custom CNN. (D to F) Training noise across datasets using ResNet18 CNN. ns, *P* > 0.05; *0.01 < *P* ≤ 0.05; ****P ≤ 0.0001.

Custom CNNs random search resulted in significantly higher F1 scores compared to training noise in case of DeepLoc and PFAM benchmark datasets (Fig. 2A and B), while the difference between F1 scores of training noise and random search on the ImmunoDB benchmark dataset was not significant (Fig. 2C). Using DeepLoc and PFAM benchmark datasets, ResNet18 CNN also produced significantly higher F1 scores during random search compared to the noise during training (Fig. 2D and E). However, the training noise of ResNet18 trained on the ImmunoDB benchmark dataset proved to be significantly higher than the F1 scores of the random search (Fig. 2F).

**Fig. 3.**
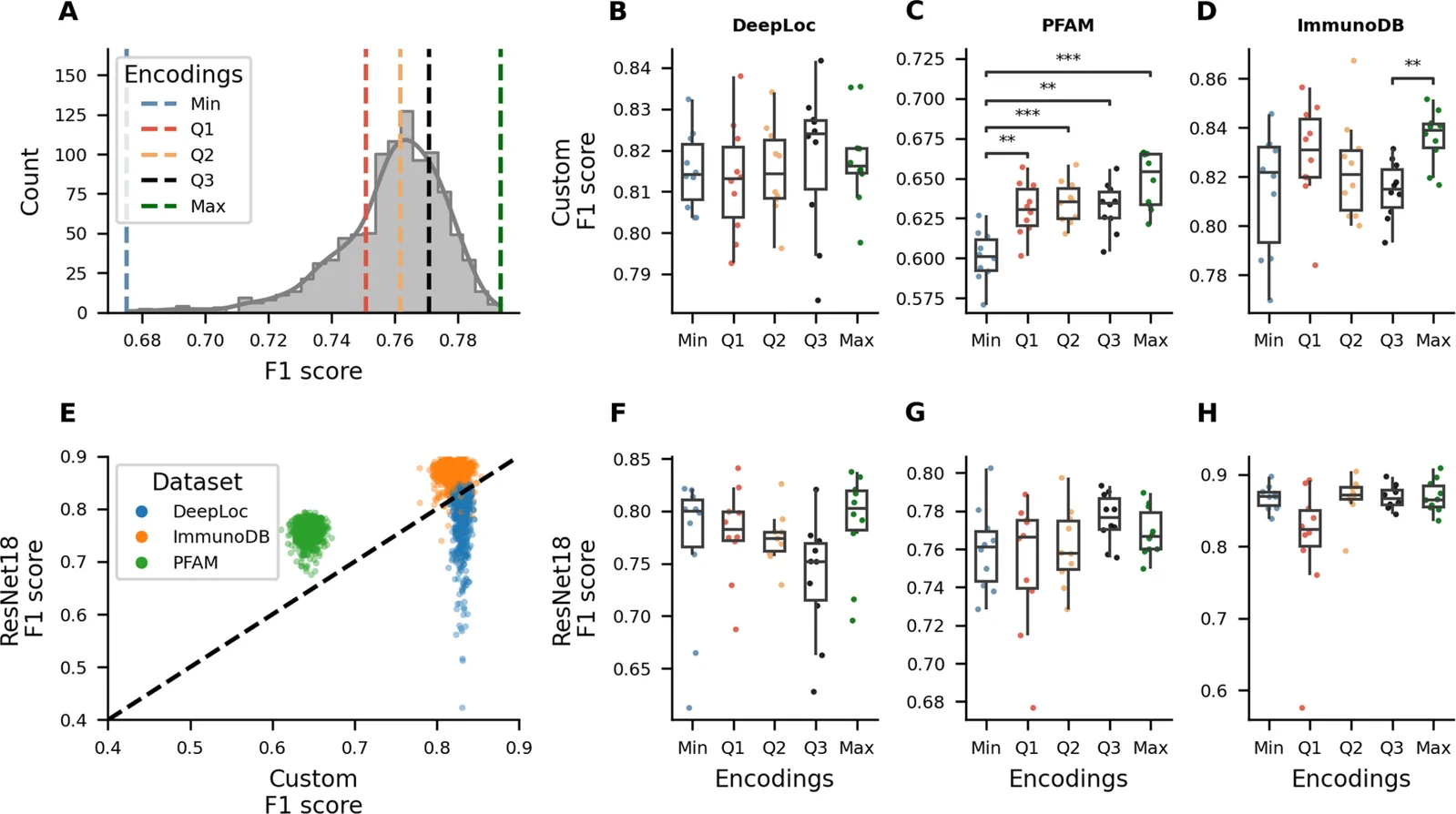
Testing different FCGR encodings through datasets and models. (A) Sample histogram displaying dedicated encodings of the PFAM dataset and ResNet18 CNN. (B to D) Cross-validation of dedicated encodings across datasets using Custom CNN. (E) Relationship between F1 scores of the Custom and ResNet18 CNNs. (F to H) Cross-validation of dedicated encodings across datasets using ResNet18 CNN. **0.001 < *P* ≤ 0.01; ***0.0001 < *P* ≤ 0.001

During the 10 fold CV of the dedicated encodings (Fig. 3A), only Custom CNN resulted in significantly different F1 scores in case of PFAM and ImmunoDB benchmark datasets (Fig. 3C and D). When Custom CNN was trained on the PFAM dataset, Min encoding yielded significantly lower F1 scores compared to all other encodings (Fig. 3C). In case of Custom CNN trained on the ImmunoDB benchmark dataset, only Q3 and Max encodings yielded significantly different F1 scores (Fig. 3D). In case of ResNet18, no significant differences were found through dedicated encodings across any of the datasets (Fig. 3F to H). Interestingly, there is no relationship found between random search results of Custom and ResNet18 CNNs in any of the benchmark datasets (Fig. 3E).

**Fig. 4.**
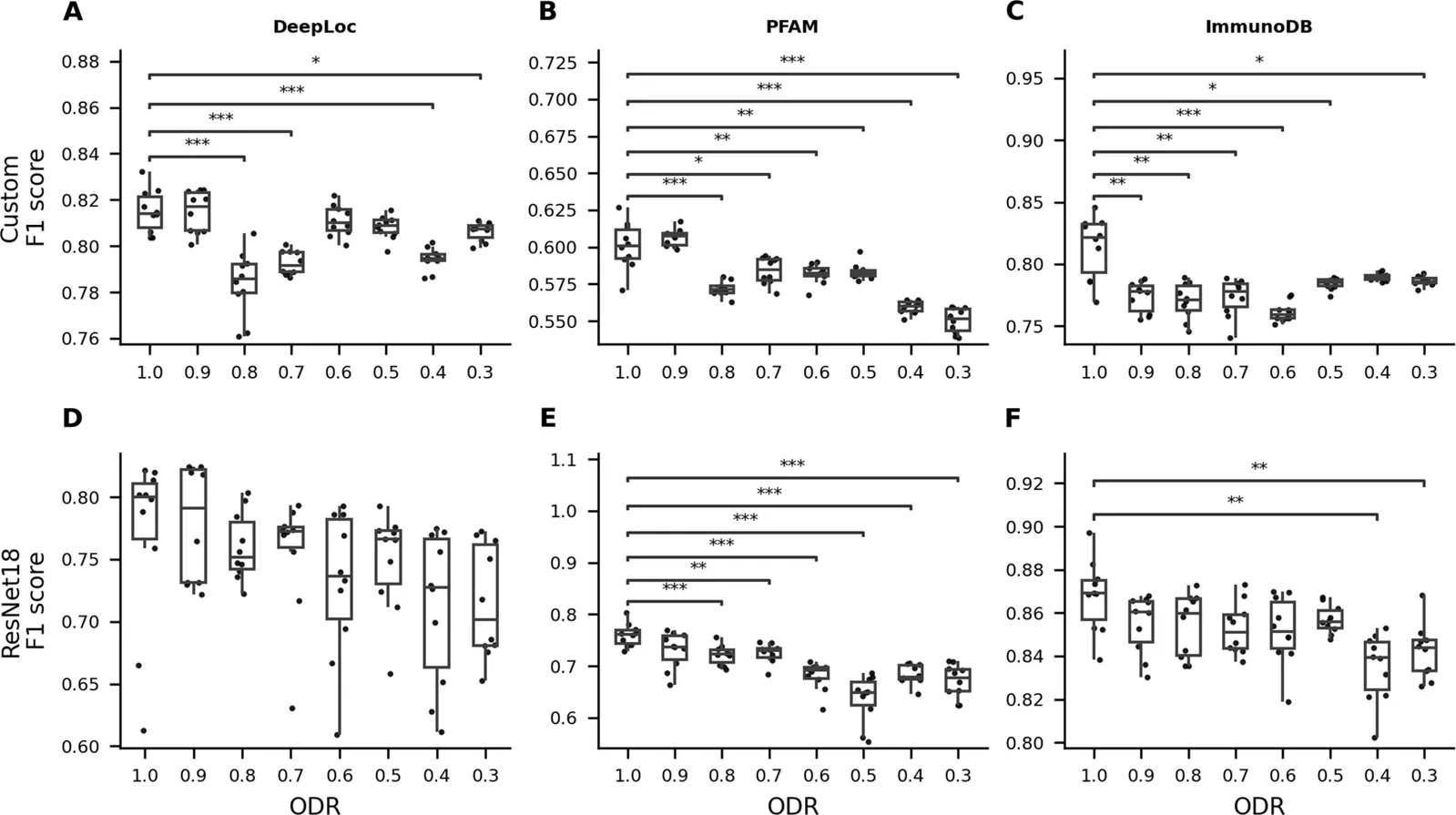
Augmentation using the mix strategy. (A to C) Augmentation across datasets using Custom CNN. (D to F) Augmentation across datasets using ResNet18 CNN. *0.01 < *P* ≤ 0.05; **0.001 < *P* ≤ 0.01; ***0.0001 < *P* ≤ 0.001.

When using mix augmentation strategy, the most frequent pattern is that the classification performance goes down as ODR decreases (Fig. 4). In case of training Custom CNN on ImmunoDB, even a slight drop in ODR (ODR = 0.9) caused a significant decrease in F1 score compared to the ODR = 1.0 case (Fig. 4C). Furthermore, a Custom CNN trained on ImmunoDB yielded significantly decreased F1 score no matter the ODR (Fig. 4C). Similarly, apart from the case ODR = 0.9, the Custom CNN trained on the PFAM benchmark dataset resulted in significantly lower F1 scores (Fig. 4B). However, the same CNN produced significantly lower F1 scores only in case of ODR = 0.8, 0.7, 0.4, and 0.3 when trained on the DeepLoc benchmark dataset (Fig. 4A). Surprisingly, none of the ODRs tested yielded different F1 scores compared to ODR = 1.0 when ResNet18 CNN was trained on the DeepLoc benchmark dataset (Fig. 4D). Except the case ODR = 0.9, ResNet18 CNN trained on the PFAM dataset resulted in a significantly decreased F1 score no matter the ODR applied (Fig. 4E). Remarkably, when ResNet18 was trained on the ImmunoDB dataset, only the cases ODR = 0.4 and 0.3 yielded a lower F1 score compared to ODR = 1.0 (Fig. 4F).

**Fig. 5.**
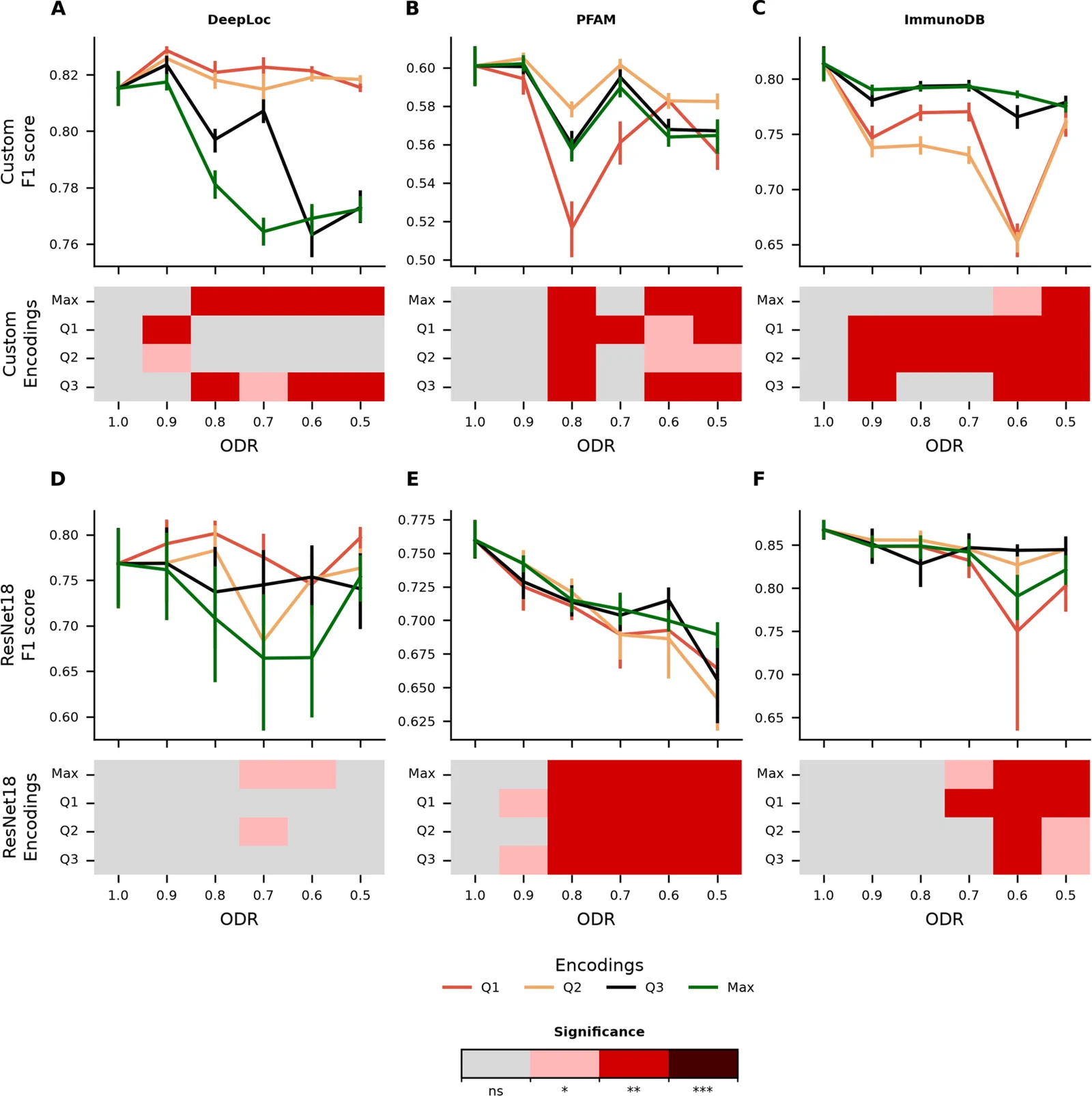
Augmentation using the single strategy. (A to C) Augmentation across datasets using Custom CNN. (D to F) Augmentation across datasets using ResNet18 CNN. Heatmaps show the significance of Kruskal–Wallis test between cross-validated F1 scores of Min encoding and other encodings at different original dataset ratios (ODRs). ns, *P* > 0.05; *0.01 < *P* ≤ 0.05; **0.001 < *P* ≤ 0.01; ***0.0001 < *P* ≤ 0.001.

With respect to the single augmentation strategy, there were cases in which classification performance did not drop regardless of the ODR used when Custom (Fig. 5A) or ResNet18 (Fig. 5D) CNNs were trained on the DeepLoc benchmark dataset. Additionally, in case of Custom CNN trained on DeepLoc Q1 and Q2 encodings with ODR = 0.9, classification performance got even better compared to ODR = 1.0 (Fig. 5A). In contrast, when Custom CNN was trained on Q1 or Q2 augmented encodings of ImmunoDB benchmark, the F1 scores significantly decreased no matter the ODR applied (Fig. 5C). Unexpectedly, when ResNet18 CNN was trained on the PFAM benchmark, after the case of ODR ≤ 0.8, the F1 scores dropped (Fig. 5E). Interestingly, there are cases where a CNN yielded better performance at lower ODR regardless of the CNN, dataset or encoding (Fig. 5B to D).

**Fig. 6.**
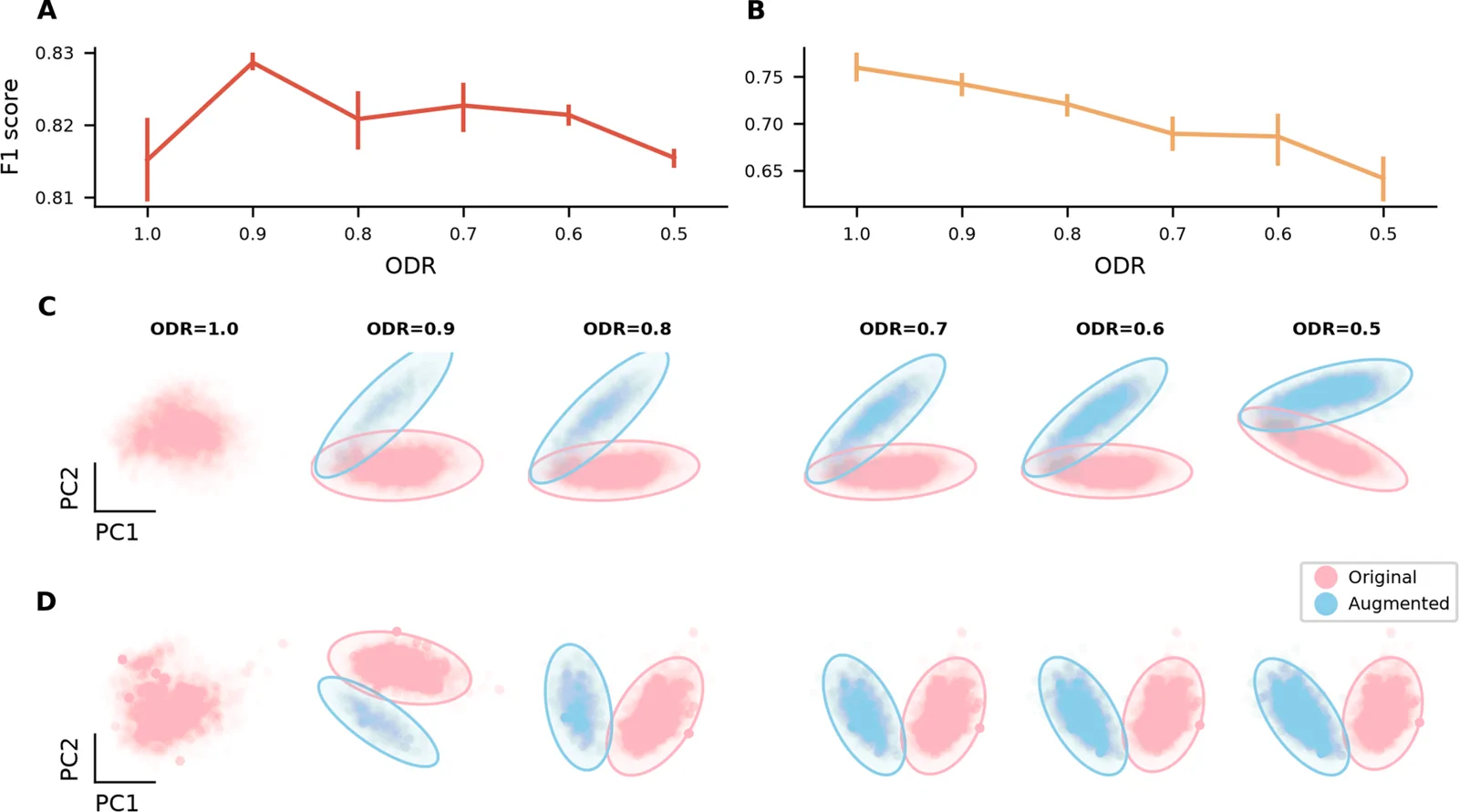
Distributional shift in sample cases as a possible explanation to single augmentation outcome. (A) DeepLoc dataset using Custom CNN and Q1 encoding. (B) PFAM dataset using ResNet18 CNN and Q2 encoding. (C) PCA of original and augmented data points during single augmentation of DeepLoc dataset using Custom CNN and Q1 encoding. (D) PCA of original and augmented data points during single augmentation of PFAM dataset using ResNet18 CNN and Q2 encoding.

**Fig. 7.**
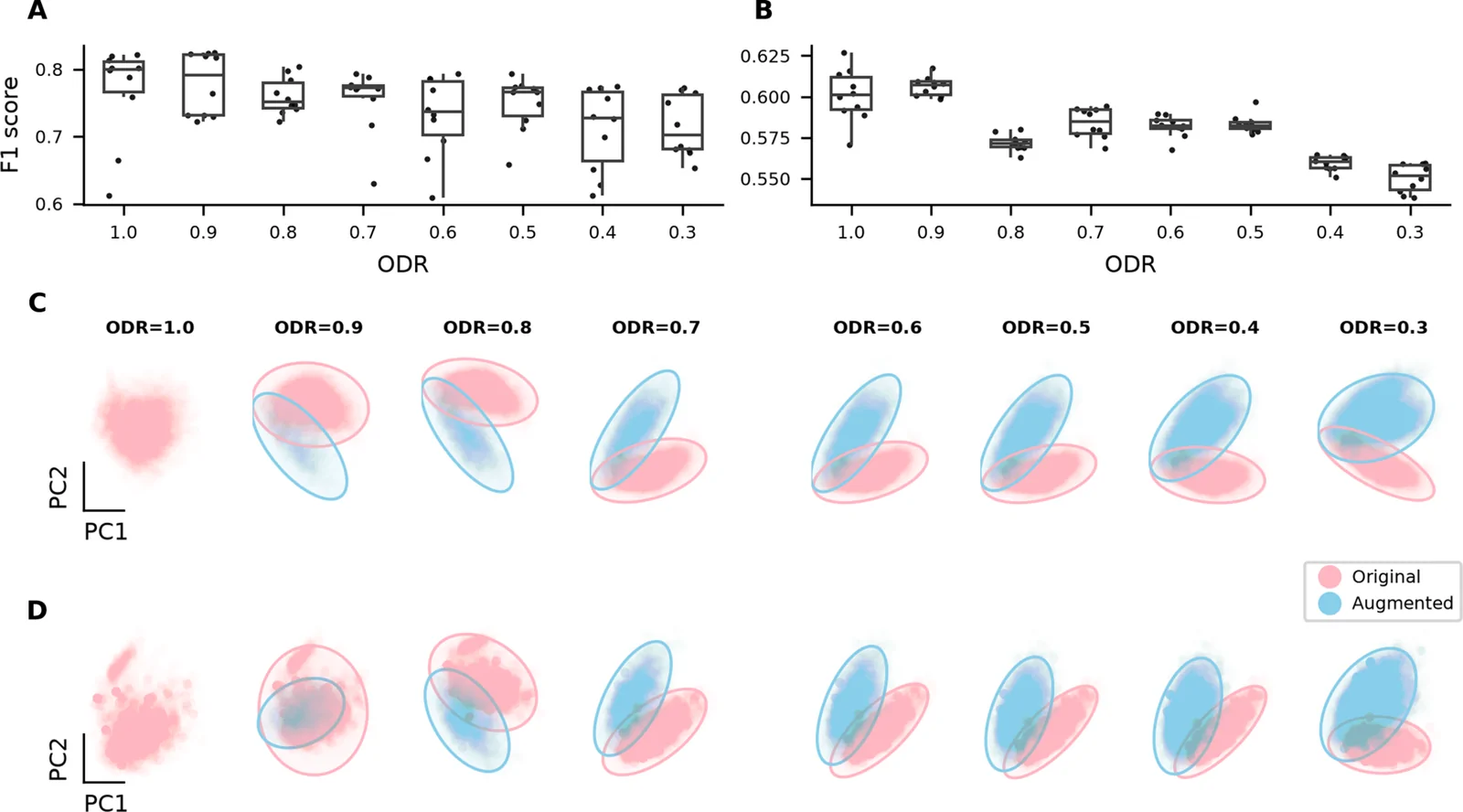
Distributional shift in sample cases as a possible explanation to mix augmentation outcome. (A) DeepLoc dataset using ResNet18 CNN. (B) PFAM dataset using Custom CNN. (C) PCA of original and augmented data points during mix augmentation with ResNet18 CNN trained on DeepLoc benchmark dataset. (D) PCA of original and augmented data points during mix augmentation with Custom CNN trained on PFAM benchmark dataset.

To see whether there is a difference between augmented and original data points, principal component analysis (PCA) was applied to the augmented FCGR data (Figs. 6 and 7). When reducing the augmented data to the first 2 principal components, the original and the augmented data points overlap to a certain extent. If there is no significant difference between F1 scores of the CNN trained on the original data and F1 scores coming from the augmented data, the overlap is stable through ODRs. This applies to cases where slight or no significant decrease could be observed in the classification performance across ODRs, such as when Custom CNN was trained on the Q1 encoding of the DeepLoc benchmark (Figs. 5A and 6A and C). In contrast, in cases where the performance dropped as ODR went down, the augmented and the original data points show clear separation based on the first 2 principal components. Such is the case when ResNet18 CNN was trained on the Q2 encoding of the PFAM benchmark dataset (Figs. 5E and 6B and D).

The same pattern could be observed in case of mix augmentation strategy (Fig. 7). When there was no difference between F1 scores through the ODRs compared to the nonaugmented F1 scores, the overlap between augmented and nonaugmented data points was stable but negligible. An example to this phenomenon was when ResNet18 CNN was trained on the DeepLoc benchmark dataset (Figs. 4D and 7A and C). On the other hand, when the overlap between original and augmented data points decreases over ODRs, the F1 scores differ significantly. This is what happened when Custom CNN was trained on the PFAM benchmark dataset (Figs. 4B and 7B and D). Interestingly, during this augmentation experiment, ODR = 0.9 yielded higher F1 scores than the F1 scores of the CNN trained on nonaugmented data (Fig. 7B). This result is supported by the PCA plot as the data points completely overlap (Fig. 7D).

## Discussion

Different vertex assignments of CGR/FCGR encoding were evaluated on classification and data augmentation downstream tasks. The vertex assignments for generating CGR encodings are rather arbitrary [24], serving visualization purposes [13] or grouped based on physicochemical properties of the proteinogenic amino acids [12,14,25,26]. Some suppose that changing the vertex assignment of CGR is not affecting downstream tasks [13]. Here, we showed that CGR permutations with different vertex assignments were used for FCGR generation and using those FCGRs to train CNN-based classifiers indeed makes a difference in classification performance as significant differences were found between F1 scores of different CGR permutations. The significant differences across vertex assignments in F1 scores make CGR/FCGR encoding a method that requires careful fine-tuning in performance critical applications. In Ref. [14], the authors performed experiments using alternative nodal orientations. However, in this paper, only 4 vertex assignments were tested in multiclass classification tasks. Additionally, all the alternative nodal orientations tested were based on physicochemical parameters (polarity and molecular weight) of amino acids. These details make our results incomparable.

Another paper [8] found that sequences’ motive composition is crucial as it creates the link between physicochemical parameters and the optimal vertex assignment used for CGR/FCGR generation. During our experiments, we found that the optimal vertex assignment is not only driven by biochemical factors. Our result indicates that finding the best alternate nodal orientation is dependent on and should be tailored to the model and dataset used. For example, ResNet18 CNN outperformed our Custom CNN on 2 of the benchmark datasets except DeepLoc. Another interesting finding that strengthens the dataset and model-dependent CGR/FCGR hypothesis is that training noise F1 scores were significantly lower compared to the search F1 scores in case of PFAM and DeepLoc but not in the case of the ImmunoDB benchmark dataset. The theory that some structural patterns captured by CGR/FCGR make certain neural network architectures better suited to particular problems has already been proposed elsewhere [27,28].

Moreover, we showed that using CGR/FCGR permutations as a data augmentation strategy is restricted to cases where moderate augmentation is needed. This is mainly because the newly introduced augmented data points form a distinct subpopulation. When ODR is high enough, meaning that the model is trained mostly on original data points, there is no significant difference between training and testing datasets. This results in classification performance comparable (not significantly different) to models trained purely on the original dataset. However, as ODR gets lower, training and testing are performed on datasets that technically are drawn from different distributions. This results in reduced performance, hence makes radical augmentation using CGR/FCGR encoding impracticable. On the other hand, there are certain situations where the augmentation potential of CGR/FCGR encodings is revealed. These are the cases where the classification performance does not get worse or even gets better, which is the ultimate goal of data augmentation [29]. In our experiments, certain CNNs during single augmentations at different ODRs, especially in the case of DeepLoc benchmark, yielded better performance than CNNs trained on the original data. From the 6 combinations of benchmark datasets and different CNNs, only ResNet CNN trained on the ODR = 0.3 mix augmented DeepLoc benchmark dataset yielded similar performance in comparison to the CNN trained on the original dataset. This finding further strengthens the dataset and model specificity of the best-suited nodal orientation for CGR/FCGR encoding. The CGR/FCGR-based augmentation was proposed by Dick and Green [14]. However, to the best of our knowledge, no research was conducted regarding the applicability of CGR/FCGR for data augmentation, which makes our research unique in this field.

## Conclusions

In this paper, the impact of alternative nodal orientations used for CGR/FCGR encodings was evaluated on protein classification performance. Additionally, these techniques were assessed from the perspective of data augmentation. We showed that different vertex assignments for CGR/FCGR result in significantly different classification performances. Moreover, we demonstrated that using alternate nodal orientations for CGR/FCGR-based data augmentation is only applicable in cases where moderate augmentation is needed. Augmenting data with CGR/FCGR to a greater degree significantly lowered classification performance in most of the cases.
